# Characterizing the Extracellular Matrix Transcriptome of Endometriosis

**DOI:** 10.1007/s43032-023-01359-w

**Published:** 2023-10-03

**Authors:** Carson J. Cook, Noah Wiggin, Kaitlin C. Fogg

**Affiliations:** 1 Bioengineering, Oregon State University, Corvallis, OR 97331 USA; 2 Computer Science, Oregon State University, Corvallis, OR 97331 USA

**Keywords:** Remodeling, Matrisome, Menstrual cycle phase, GEO

## Abstract

In recent years, the matrisome, a set of proteins that make up the extracellular matrix (ECM) or are closely involved in ECM behavior, has been shown to have great importance for characterizing and understanding disease pathogenesis and progression. The matrisome is especially critical for examining diseases characterized by extensive tissue remodeling. Endometriosis is characterized by the extrauterine growth of endometrial tissue, making it an ideal condition to study through the lens of matrisome gene expression. While large gene expression datasets have become more available and gene dysregulation in endometriosis has been the target of several studies, the gene expression profile of the matrisome specifically in endometriosis has not been well characterized. In our study, we explored four Gene Expression Omnibus (GEO) DNA microarray datasets containing eutopic endometrium of people with and without endometriosis. After batch correction, menstrual cycle phase accounted for 53% of variance and disease accounted for 23%; thus, the data were separated by menstrual cycle phase before performing differential expression analysis, statistical and machine learning modeling, and enrichment analysis. We established that matrisome gene expression alone can effectively differentiate endometriosis samples from healthy ones, demonstrating the potential of matrisome gene expression for diagnostic applications. Furthermore, we identified specific matrisome genes and gene networks whose expression can distinguish endometriosis stages I/II from III/IV. Taken together, these findings may aid in developing future *in vitro* models of disease, offer insights into novel treatment strategies, and advance diagnostic tools for this underserved patient population.

**Supplementary Information:**

The online version contains supplementary material available at 10.1007/s43032-023-01359-w.

## Introduction

Endometriosis affects approximately 10–15% of people who menstruate and is characterized by the growth of ectopic endometrium [1–4]. This can be associated with chronic, sometimes debilitating, pain, infertility, and other dysfunction of reproductive organs [1, 3, 5]. While the underlying cause of endometriosis remains unknown, tissue remodeling is critical to the pathogenesis and progression of this disease [6, 7]. Tissue remodeling is a complex and dynamic process, involving both extracellular matrix (ECM) deposition as well as ECM degradation [8]. While individual components of the ECM and ECM-affiliated cytokines have been subject to investigation, it is crucial to recognize that the ECM itself along with its affiliated proteins forms a complex interconnected network comprising over 1000 genes, collectively known as the matrisome [9]. Thus, a holistic yet targeted evaluation of the entire endometriosis matrisome holds the potential to elucidate specific microenvironmental cues involved in the underlying pathogenesis as well as perpetuation of endometriosis.

Though endometriosis has been shown to have a strong association with heredity and family clustering, it is not hereditary in a predictable Mendelian manner [2, 4]. Transcriptomics analyses, which quantify and assess gene expression in disease and healthy tissue, are well-suited for characterizing gene expression in endometriosis pathophysiology. However, only one large-scale transcriptomic analysis has been performed using DNA microarrays to study endometriosis, assessing global gene expression and focusing on immune infiltration [4]. To our knowledge, no existing studies have performed a targeted analysis of matrisome gene expression in endometriosis using large gene expression datasets of endometriosis tissue samples. Thus, the goal of this study was to establish the significance of the matrisome in characterizing endometriosis and identify key matrisome components which have inferential value with respect to the initiation of endometriosis and distinguishing endometriosis I/II from III/IV.

In this study, we unified publicly available whole transcriptome microarrays from normal and endometriosis samples of eutopic endometrium. We employed a variety of statistical and machine learning methods to explore dysregulation of genes in endometriosis and identify the matrisome genes, gene networks, gene ontology (GO) terms [10], and pathways [11] involved in endometriosis dynamics. We found that matrisome gene expression effectively stratified endometriosis and normal tissue and that ECM-related GO terms were highly enriched among differentially expressed matrisome genes. Additionally, we found that the menstrual cycle phase accounted for over a third of the matrisome gene expression variance; thus, we needed to separate the data by menstrual cycle phase before performing differential expression analysis, statistical and machine learning modeling, and enrichment analysis. From these approaches, we identified matrisome genes and gene networks with inferential significance to separate endometriosis stages I/II from III/IV.

## Materials and Methods

### Data Sources and Preprocessing

All data preprocessing was done using the R programming language. [12]

### Gene Expression Omnibus Data

The Gene Expression Omnibus (GEO) database (http://ncbi.nlm.nih.gov/geo/) was accessed to retrieve four datasets using the same search criteria, and subsequent filtration methods, as Poli-Neto et al. [4] The four datasets which were retrieved were from GSE4888 [13], GSE6364 [14], GSE7305 [15], and GSE51981 [16]. However, the data corresponding to GSE7305 were excluded from our analyses due to the samples being paired (disease and normal tissue samples taken from the same patient) and the tissue samples were collected from the ovaries. We also included an additional dataset of only healthy endometrium, GSE29981, that was not included in the original search criteria, specifically for the purpose of building a training set for our logistic regression classification model. In consideration of the distinct phenotypic and genotypic characteristics associated with different stages of endometriosis, we stratified the endometriosis samples into two groups: endometriosis stages I–II and III–IV, respectively. Additionally, samples with unknown menstrual cycle phase or ambiguous histology readings were excluded from our analyses. A summary of clinical information regarding each dataset is included in Supplemental Table 1. As this is an *in silico* study, the quality of sample collection cannot be assured by the authors, since the authors have access only to the data of the public repository.

Each of these datasets was made up of samples assessed using the Affymetrix Human Genome U133 Plus 2.0 Array (HG-U133 Plus 2, Affymetrix, Santa Clara, CA). [4] The data were then loaded and normalized via the robust multiarray average method (RMA) using the *Affy* package [17–19]. Finally, the data were batch corrected using the *comBat* function from the *sva* package [20], using GSE4888 as the reference batch. This was the approach that yielded the best empirical results in terms of removing batch effects (along with batch interactions) and the most variance attributable to disease condition and menstrual cycle phase. The weighted proportion of variance for the effects factors of interest was computed using principal variant component analysis (PVCA), which combines principal component analysis (PCA) and variance component analysis (VCA) to determine the amount of variance in the data attributable to specified variables [21]. The *pvcaBatchAssess* function from the *pvca* package was used with the parameter *threshold* assigned a value of 0.6. This function allowed us to assess the relative amount of variance, divided among factors of interest using principal components, which explained 60% of the variance in the data [22]. Clinical data were retrieved by downloading the series matrix files, loading them using the *getGEO* function from the *GEOquery* package [23], and then performing necessary data cleaning.

### The Matrisome Database

The human matrisome database, compiled by Naba et al., was retrieved from their online repository (http://matrisomeproject.mit.edu/other-resources/human-matrisome) on 2020/07/21 [24]. Genes classified as “retired” were filtered out, yielding a master list of 1027 genes, 964 of which were present in the GEO datasets. Of the genes in the combined dataset, the divisions consisted of “Core matrisome” ($n=258$) and “Matrisome-associated” ($n=706$). Matrisome categories included: collagens, ECM glycoproteins, ECM regulators, ECM-affiliated proteins, proteoglycans, and secreted factors (Table 1).

**Table 1:** Matrisome category counts in master list and in dataset. Counts of matrisome genes in each matrisome category in the matrisome master list and in our dataset. Of the 1027 non-retired matrisome genes in the human matrisome master list, 964 were tested for using the Affymetrix Human Genome U133 Plus 2.0 Array chip

Division	Category	Master list count	Dataset count
Core matrisome	Collagens	44	44
Core matrisome	ECM glycoproteins	195	179
Core matrisome	Proteoglycans	35	35
Matrisome-associated	ECM regulators	238	230
Matrisome-associated	ECM-affiliated proteins	171	151
Matrisome-associated	Secreted factors	344	325

### Dimensionality Reduction

Principal component analysis (PCA) was performed using the *prcomp* function from the *stats* package in *R* [12]*.* PCA was used to explore batch and biological effects in the data.

### Unsupervised Analysis

Hierarchical clustering was conducted to group samples based on similarities in their matrisome expression, without any reference to the corresponding clinical labels. The matrisome expression data were standardized to ensure that the variability in expression levels between different genes did not affect the clustering process. A clustermap was then created using a complete linkage method, which works by minimizing the furthest Euclidean distance between observations from different clusters. Although the clustering itself was unsupervised, we incorporated two sidebars alongside the heatmap to display the clinical condition and the phase of menstrual cycle associated with each sample. This was done to allow for a post-hoc exploration of any patterns that may emerge between these clinical labels and the clusters identified by the analysis.

### Stratification of Endometriosis and Normal Tissue

A training dataset was built from GEO datasets GSE4888, GSE51981, and GSE29981 and the GSE6364 dataset was held out and used as a test set (Supplemental Table 1). Within each phase, elastic net logistic regression models were trained on the Robust Multi-array Average (RMA) matrisome data [25]. Elastic net regression utilizes the objective function ${J}_{EN}\left(\beta \right)=J\left(\beta \right)+{\lambda }_{1}{\| \beta \| }_{1}+{\lambda }_{2}{\| \beta \| }_{2}$, where $J\left(\beta \right)$ is a less complex loss function, and the parameters ${\lambda }_{1}$ and ${\lambda }_{2}$ control the proportion of L^1^ (lasso) and L^2^ (ridge) regression penalization to use. Within each phase, these models were trained to classify samples as endometriosis as normal or endometriosis tissue. Model performance was measured using balanced classification accuracy [26]. This scoring method utilizes observation weights defined according to ${w}_{c}=\frac{n}{C\cdot {n}_{c}}$, where ${w}_{c}$ is the weight assigned to observations from class $c$, $n$ is the total number of observations, $C$ is the total number of classes (factor levels of the response), and ${n}_{c}$ is the number of observations in class $c$. [27] Observation weights sum to 1, ensuring that models are penalized equally for bad performance in any class regardless of the imbalanced representation of classes. Using sequential model-based optimization [28], these models were optimized based on 5-fold cross-validation scores. All features (genes) were standardized to have a mean of 0 and a standard deviation of 1. The *scikit-learn* implementation of logistic regression was used [27], and sequential model-based optimization was performed using *gp_minimize* from *scikit-optimize* [29]*.* These packages are open source and available for the Python programming language, which was used for this portion of the study.

### Differential Gene Expression Analysis

Differential gene expression (DGE) analysis was conducted on the full set of Robust Multi-array Average (RMA) normalized gene expression counts using the *limma* package in *R* [30]*.* All genes with expression units larger than $\left(50\right)$ (*Affy*’s *rma* function produces results in ${log}_{2}$) in at least 25% of samples were considered sufficiently expressed. A permissive expression cutoff was chosen so that matrisome genes were filtered at similar rates in each category to genes overall (Supplemental Table 2). DGE analysis was then performed by phase, and genes with a log fold-change of $\left(1.5\right)$ and adjusted $p$-value less than 0.05 were considered differentially expressed. Adjusted $p$-values were computed using the Benjamini-Hochberg false discovery adjustment method.

### Univariable and Multivariable Statistical Analyses

Several univariate and multivariable statistical analyses were performed on the RMA normalized gene expression data (endometriosis samples only). The stage-wise DGE analysis was performed using all genes to allow for better estimation of global parameters. The other analyses were performed on the matrisome genes alone. False discovery rates were estimated for statistical tests using computed $q$-values [31]. Similar to the endometriosis versus normal tissue DGE analysis, the data were stratified by phase for each analysis, yielding separate results for each menstrual cycle phase.

### Stage-Wise Differential Gene Expression Analysis

Differential gene expression analysis was performed between endometriosis samples from the two different endometriosis stages in the dataset, endometriosis I/II and endometriosis III/IV. The same minimum expression threshold as the endometriosis versus normal DGE analysis was used. This analysis was performed on the set of all genes, at which point the results were filtered to include only matrisome genes. Differential expression was determined using the same adjusted $p$-value and log fold-change cutoffs as the endometriosis versus normal DGE analysis. The *limma* package was used for this analysis.

### Point-Biserial Correlation

Point-biserial correlation is mathematically equivalent to the Pearson correlation between a continuous and dichotomous variable [32]. Endometriosis stage was coded as an indicator variable, with a value of 1 corresponding endometriosis stages III/IV and 0 corresponding to endometriosis stages I/II. Genes were deemed significant if their Student asymptotic $q$-values were below the significance threshold ($q<0.05$). [33] Student asymptotic $p$-values were computed using the *corPvalueStudent* function from the *WGCNA* package and then adjusted.

### Endometriosis Stage-Predictive Penalized Logistic Regression

Multivariable L^1^ penalized logistic regression models were fit to the endometriosis data in each phase. The models were fit using the *glmnet* package in *R* [34]*.* The models were optimized for parsimony which is done by tuning the value of $\lambda$ in the L^1^ penalty objective function ${J}_{{L}^{1}}\left(\beta \right)=J\left(\beta \right)+\lambda {\| \beta \| }_{1}$, where $J\left(\beta \right)$ is some simpler loss function (misclassification rate, for example) and ${\| \beta \| }_{1}$ is the L^1^ norm of the model parameters. The models were fit using a 1-dimensional grid search over values of $\lambda$, and the least parsimonious model (smallest value of $\lambda$) which scored within 1 standard error of the best performing model was selected for each phase. This was done to avoid using models which selected too few matrisome genes, since these results were then filtered to include only DEMGs. The models were fit using 5-fold cross-validation using the *cv.glmnet* function from the *glmnet* package with arguments *family* set to “binomial” and *type.measure* set to “class.” Balanced class-weighting (as in the endometriosis/normal stratification models) was used to ensure models did not favor performance in only the majority class.

### Weighted Gene Correlation Network Analysis

Univariable and multivariable analyses assessed a gene’s independent association with endometriosis stage or a gene’s ability to serve as a proxy for a set of other co-expressed genes. In contrast, weighted gene correlation network analysis (WGCNA) identified genes which, as a cluster, demonstrated a significant link to delineating endometriosis I/II from III/IV [33]. This was achieved by establishing unsigned gene co-expression modules, where co-expression is estimated using unsigned topological overlap measures [35]. Each module was then represented using the module’s eigengene, which was the first principal component representation of the gene expression of all genes assigned to that module [33]. These eigengenes were then be correlated with endometriosis stage. Finally, a module’s constituent genes were identified as significant based on correlation tests with their respective eigengenes.

WGCNA was performed according to the instructions provided by the package authors [36]. First, the data were stratified by phase. Then, a topological overlap measure matrix was constructed over the matrisome gene expression values using a minimum soft power threshold which yielded a scale-free topological overlap metric (TOM) greater than 0.8 [35], representing a gene-wise estimate of unsigned co-expression among matrisome genes. Hierarchical clustering was then performed on this matrix, and modules with a correlative distance of 0.25 or less (i.e., module correlation of 0.75 or more) merged. The modules found using this method were then related to endometriosis severity via point-biserial correlation. Matrisome genes that belonged to a module which was significantly correlated with endometriosis stage (Student asymptotic $q<0.05$) and showed significant correlation with their respective module eigengenes (Student asymptotic $p<0.05$) were deemed significant. All WGCNA was performed using *R* code and functions from the *WGCNA* package in *R.* [33]

### Enrichment Analysis

Gene set and pathway enrichment analyses were performed using the *clusterProfiler* package in *R*. [37] For gene set enrichment analysis, the function *enrichGO* was used to find enriched gene ontologies (GO) among significant genes [38]. Subsequently, the *simplify* function was utilized to group similar GO terms and select representative terms for each group, thereby simplifying the interpretation of results. For pathway enrichment analysis, the function *enrichKEGG* was used to find KEGG pathways that were enriched among significant genes [11]. Gene function and pathway significance was determined based on the $q$-value reported in the results of each function ($q<0.05$).

## Results

### Sources of Variance

**Fig. 1:**
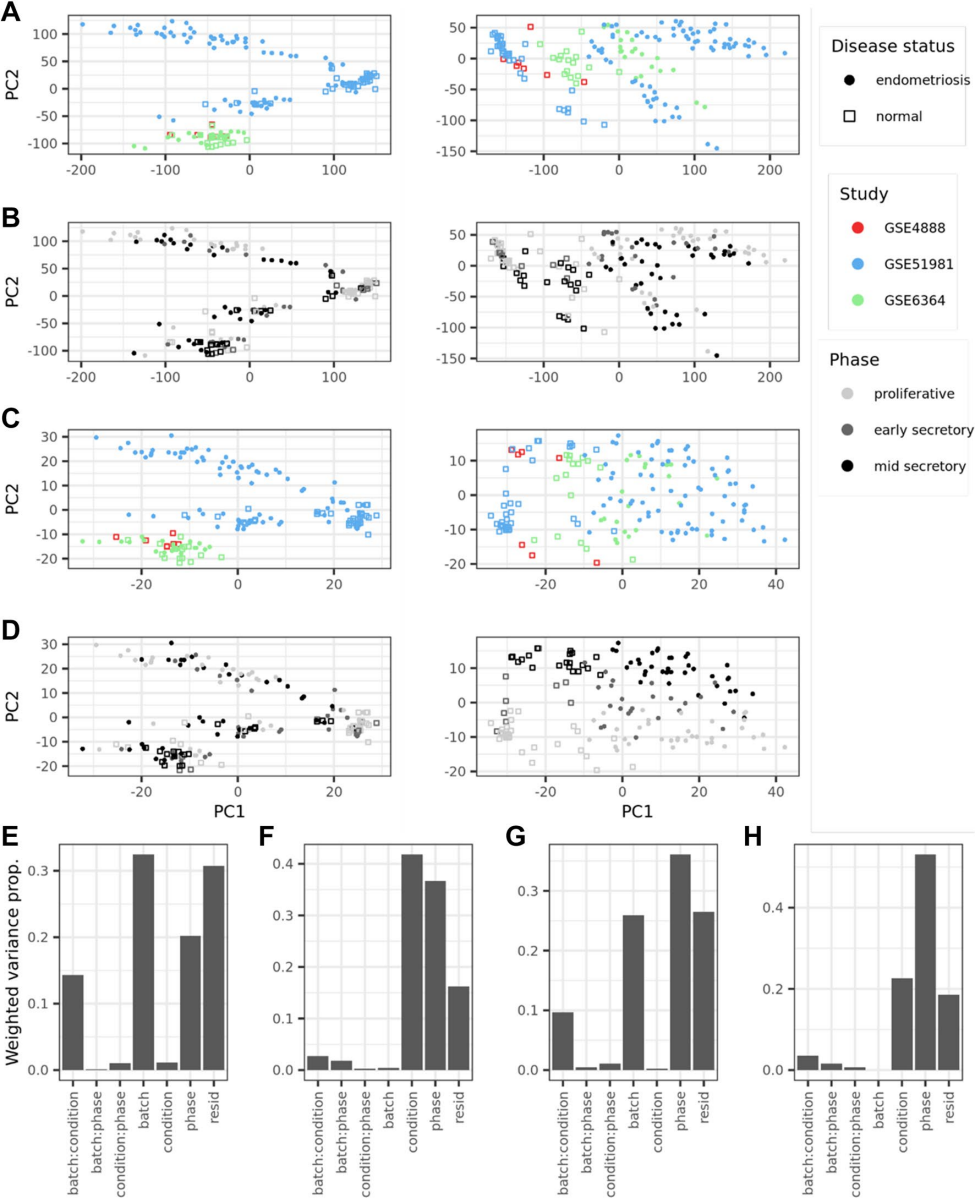
Sources of variance within the data. Each clinical study is distinguished by color (GSE4888, red; GSE51981, blue; and GSE6364, green), each menstrual cycle phase is distinguished by shade (proliferative, light gray; early-secretory, dark gray; and mid-secretory, black), and disease status is distinguished by shape (normal, open squares; endometriosis, closed circles). **A** Principal component analysis (PCA) of the full gene expression data (${n}_{\mathrm{genes}}=\mathrm{21,407}$) before (left) and after (right) batch correction. Percent variance explained: left (PC1, 33.6%; PC2, 24.3%), right (PC1, 49.4%; PC2, 9.3%). **B** PCA of the full gene expression data before (left) and after (right) batch correction. Percent variance explained: left (PC1, 33.6%; PC2, 24.3%), right (PC1, 49.4%; PC2, 9.3%). **C** PCA of the matrisome gene expression data (${n}_{\mathrm{mat}.\hspace{0.25em}\mathrm{genes}}=964$) before (left) and after (right) batch correction. Percent variance explained: left (PC1, 27.5%; 20.6%), right (PC1, 41.6%; PC2, 10.0%). **D** PCA of the matrisome gene expression data before (left) and after (right) batch correction. Percent variance explained: left (PC1, 27.5%; 20.6%), right (PC1, 41.6%; PC2, 10.0%). **E** Principal variant component analysis (PVCA) of global gene expression before batch correction. For PVCA, the $x$-axis corresponds to the factors of interest and their linear interaction terms. **F** PVCA of global gene expression after batch correction. **G** PVCA of matrisome gene expression before batch correction, **H** PVCA of matrisome gene expression after batch correction

To examine the sources of variance in our dataset, we used principal component analysis (PCA). Because the data we used came from three different clinical studies, we assessed the level of variance attributable to technical differences in the data collection processes. To correct for these sources of technical variance, often called “batch effects,” we performed batch correction using an empirical Bayes method (Fig. 1). [39] The data used in our study consisted of tissue samples from the proliferative, early-secretory, and mid-secretory phases of the menstrual cycle from patients with and without a clinical diagnosis of endometriosis.

For the global gene expression data (${n}_{\mathrm{genes}}=\mathrm{21,407}$), before batch correction the first principal component captured 34% of the variance and appeared to separate samples mostly by disease status, whereas the second component captured 24% of the variance and appeared to primarily separate samples by study. After batch correction, the first component explained 49% of the variance in the data and clearly separated samples by disease status. The second component explained only 9% of the variance and seemed to somewhat separate samples by menstrual cycle phase (Fig. 1A, B). When we examined matrisome gene expression alone (${n}_{\mathrm{mat}.\mathrm{genes}}=964$), before batch correction the first principal component captured 28% of the variance and appeared to separate samples mostly by disease status, while the second component captured 21% of the variance and separated samples primarily by study. After batch correction, the first component accounted for 42% of the variance and separated samples by disease status, while the second component explained 10% of the variance and appeared to mostly separate samples by menstrual cycle phase (Fig. 1C, D). Overall, the effects of batch correction can be seen by comparing the clustering of samples by study number on the left column before clustering (Fig. 1A, C) compared to the clustering of samples by menstrual phase and disease status in the right column (Fig. 1B, D).

Principal variant component analysis (PVCA) was then used to reduce the gene expression data to the first principal components which accounted for 60% of the variance, then evaluate relative proportions of variance within these principal components due to clinical study, disease status, menstrual phase, and their interactions. PVCA was performed before and after batch correction for global (Fig. 1E, F) and matrisome (Fig. 1G, H) gene expression. Before batch correction, batch (study) and batch interaction terms accounted for approximately 47% of the variance in the global gene expression data and 36% in the matrisome gene expression data. After batch correction, these percentages were reduced to approximately 5% in both the global and matrisome gene expression data. We found that disease status accounted for the most variation in the batch corrected global expression data (42%), closely followed by menstrual cycle phase (37%). For the batch corrected matrisome expression data, menstrual cycle phase accounted for the most variation (53%), followed by disease status (23%). The high level of variance attributed to menstrual cycle phase in the matrisome gene expression data was unsurprising, given that the endometrium is highly dynamic and heterogeneous between phases of the menstrual cycle. [13, 40]

### Stratification by Menstrual Phase

**Fig. 2:**
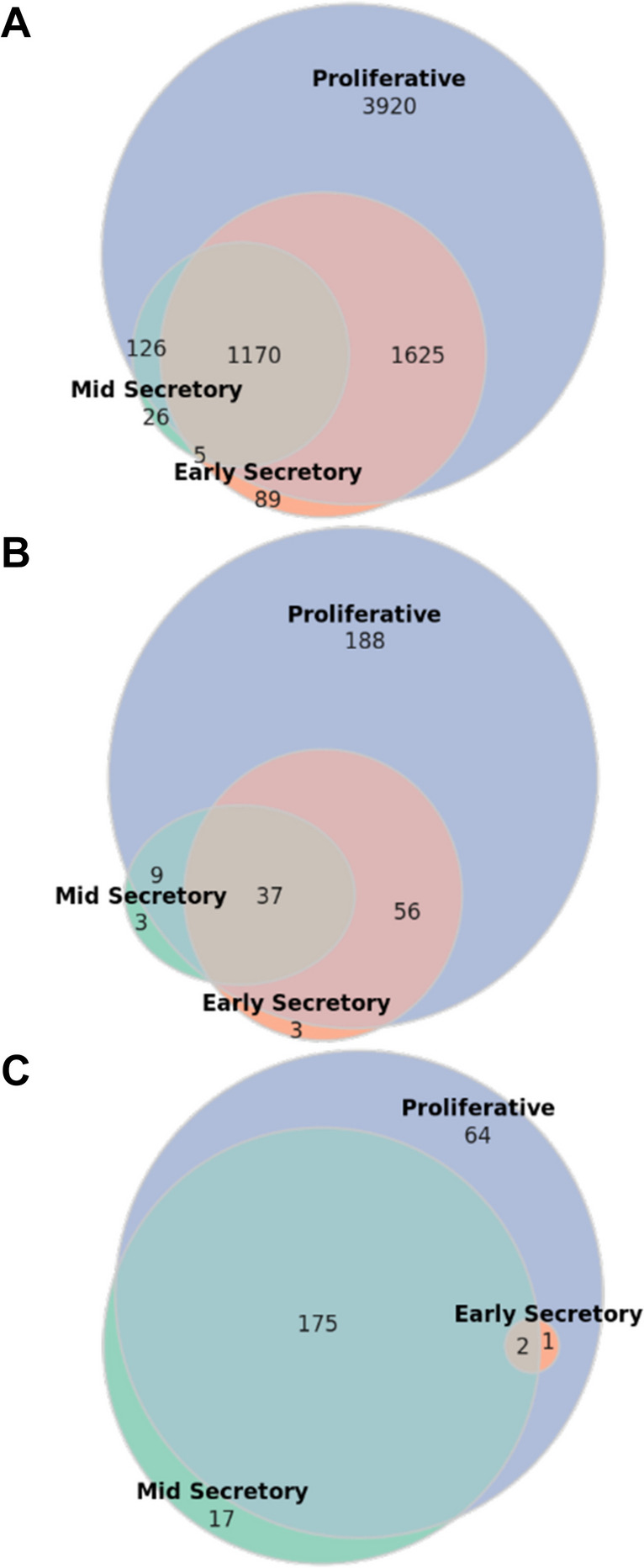
Overlaps of significant genes between phases. Inter-phase overlaps within the set of **A** genes which were differentially expressed between normal and endometriosis tissue (DEGs) ($n=6961$), **B** matrisome genes which were differentially expressed between normal and endometriosis tissue (DEMGs) ($n=296$), and **C** DEMGs which were significant with respect to endometriosis stage ($n=259$)

The demonstration of extensive variance attributable to menstrual cycle phase prompted us to adopt a similar approach to Poli-Neto et al. and stratify the data by menstrual cycle phase before performing our differential expression analysis, statistical and machine learning modeling, and enrichment analysis [4]. However, in each of these analyses, it became apparent that the differentially expressed and otherwise significant matrisome genes in the early- and mid-secretory phases were almost entirely subsets of those found to be differentially expressed or significant in the proliferative phase (Fig. 2). Furthermore, when there was overlap between phases, the directionality of significant matrisome gene influence (e.g., fold-change sign) was also virtually identical between phases for all analyses. Only 1 of 6961 unique DEGs was found to be differentially expressed in endometriosis in an inconsistent direction between phases, and only 1 of 259 DEMGs later found to be significantly related to endometriosis stage was directionally inconsistent between phases (Supplemental Table 3). For this reason, we decided to conduct the various analyses separately within each phase, then perform a union operation on the results of the various analyses—pooling all unique genes significant in any phase—before conducting enrichment analyses.

### Differentially Expressed Genes Between Endometriosis and Normal Uterine Tissue Samples

**Fig. 3:**
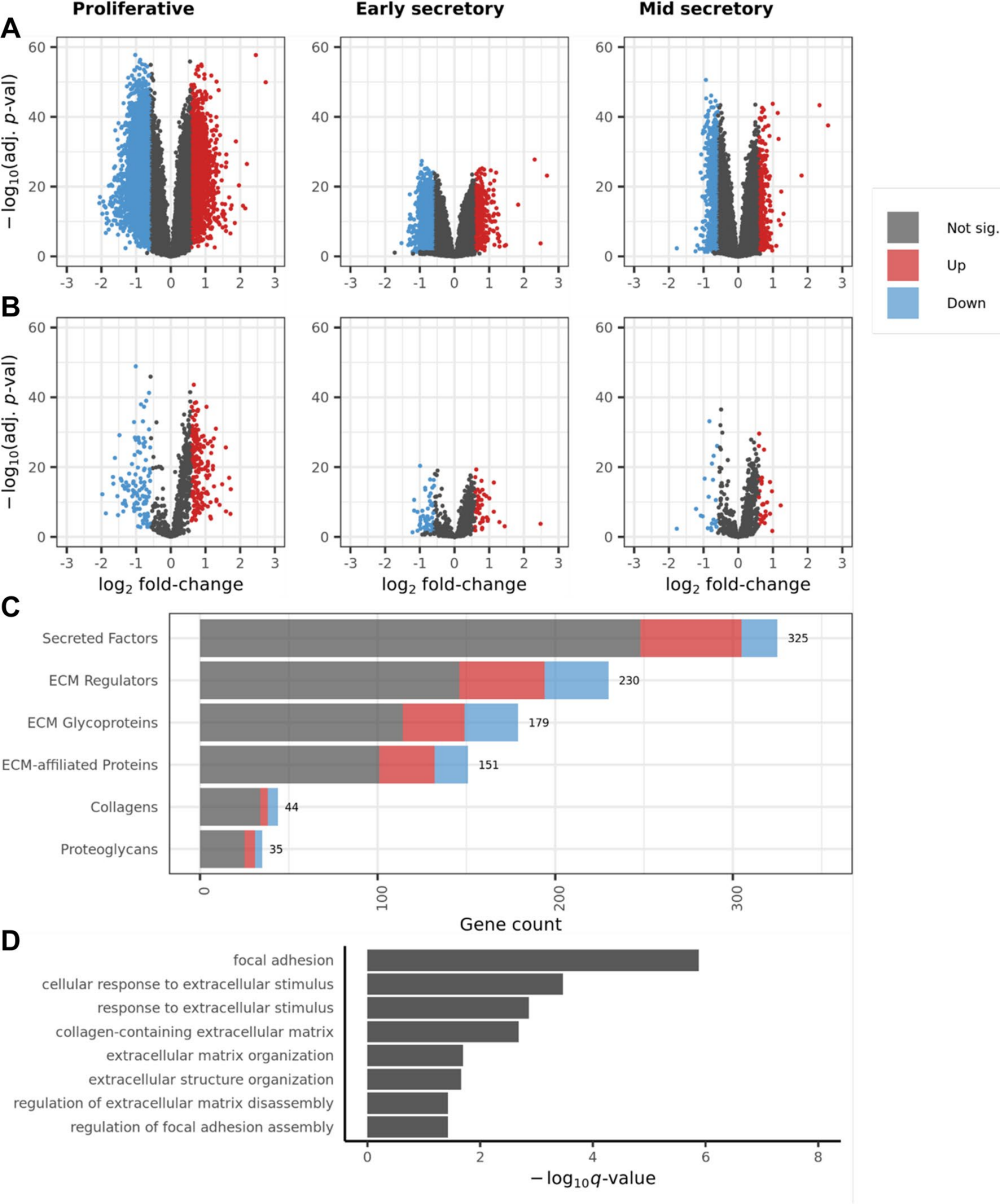
Differential gene expression within each phase and functional enrichment analysis of combined results. Differentially expressed genes in each phase among **A** all genes and **B** matrisome genes. **C** Breakdown of differentially expressed matrisome genes (union of results in all phases) by matrisome category. **D** Results of functional enrichment analysis for all genes, with respect to ECM-related gene functions. Gene counts in dataset: all genes (${n}_{\mathrm{genes}}=\mathrm{21,415}$) and matrisome genes (${n}_{\mathrm{mat}.\hspace{0.25em}\mathrm{genes}}=964$)

To investigate the importance of the matrisome in characterizing endometriosis, we performed differential gene expression (DGE) analysis on the full set of genes in the dataset (${n}_{\mathrm{genes}}=\mathrm{21,407}$), comparing endometriosis samples (${n}_{\mathrm{proliferative}}=35$, ${n}_{\mathrm{early}-\mathrm{secretory}}=24$, ${n}_{\mathrm{mid}-\mathrm{secretory}}=37$) to normal tissue samples (${n}_{\mathrm{proliferative}}=28$, ${n}_{\mathrm{early}-\mathrm{secretory}}=9$, ${n}_{\mathrm{mid}-\mathrm{secretory}}=20$), examined differential expression rates among matrisome genes (${n}_{\mathrm{mat}.\mathrm{genes}}=964$), and performed functional enrichment analysis on the full list of differentially expressed genes (DEGs) to observe whether ECM-related gene ontology (GO) terms were enriched. The DGE analysis was performed separately for samples in each phase, and 6841, 2889, and 1327 differentially expressed genes (DEGs) were found in the proliferative, early-secretory, and mid-secretory phases, respectively (Fig. 3A and Table 2). Next, we filtered these results to include only the matrisome genes and identified 290, 96, and 49 differentially expressed matrisome genes (DEMGs) in the proliferative, early-secretory, and mid-secretory phases, respectively (Fig. 3B and Table 2). These data demonstrate that global gene expression and matrisome gene expression were equally upregulated and downregulated in endometriosis samples compared to healthy samples and that samples from the proliferative phase demonstrated the greatest amount of dysregulation compared to samples from the early- and mid-secretory phases.

**Table 2:** Differentially expressed gene counts. Counts and percentages of differentially expressed (DE) genes among all genes ($n=\mathrm{21,415}$) and matrisome genes ($n=964$) within phases and after pooling (computing union of) results for each phase. Includes counts of upregulated and downregulated genes for each phase and among all phases

Phase	Total DE	% DE	Upregulated	Downregulated
*All genes* (*n* = 21,407)				
Proliferative	6841	32%	2585	4256
Early-secretory	2889	13%	493	2396
Mid-secretory	1327	6%	312	1015
Union of phases	6961	33%	2603	4357
*Matrisome genes* (*n* = 964)				
Proliferative	290	30%	179	111
Early-secretory	96	10%	44	52
Mid-secretory	49	5%	29	20
Union of phases	296	31%	181	115

Next, we performed a set union of DEGs between phases (yielding a set of all genes which were differentially expressed in one or more phases) to compare the rates of differential expression in any phase between genes overall and matrisome genes. The phase-union set of DEGs contained approximately 33% of the total set of genes, while the phase-union set of DEMGs contained approximately 31% of the total matrisome genes (Table 2). The phase-union DEMGs were then stratified by their respective matrisome categories, and we found that ECM glycoproteins and ECM regulator were differentially expressed at higher rates than the full set of genes. In contrast, ECM-affiliated proteins were differentially expressed at the same rate as the full set of genes, and proteoglycans, secreted factors, and collagens were differentially expressed at lower rates (Fig. 3C). A total of 6961 unique DEGs were contained in the phase-union DEG list while 296 unique DEMGs were contained in the phase-union DEMG list (Supplemental Table 4).

It was observed that both the DEGs (global gene expression) and DEMGs (matrisome gene expression) in the early- and mid-secretory phases were almost entirely subsets of those found to be differentially expressed in the proliferative phase. In addition, all overlapping DEGs shared by each phase were differentially expressed in the same direction, except for *ATP12A*, which was upregulated in proliferative stage samples but downregulated in mid-secretory samples. Due to the fact that *ATP12A* is not defined as a matrisome gene, no DEMGs which were shared by each phase had disagreement in differential expression direction. Furthermore, DEGs and DEMGs in the mid-secretory phase seemed to be almost entirely a subset of the DEGs and DEMGs in the early-secretory phase. Taken together, these data indicate that the maximum dysregulation between endometriosis and normal uterine tissue occurs in the proliferative phase. It also implies that the dysregulation which occurs in the early- and mid-secretory phases is a reduced form of the dysregulation which occurs in the proliferative phase. After making this observation, we defined our final set of DEGs to be this union list, which contained genes that were differentially expressed in at least one phase of the menstrual cycle in tissue from patients with endometriosis compared to those without endometriosis across all menstrual phases. This approach identified the genes that were overall dysregulated in endometriosis regardless of menstrual phase. This final list was then used to define DEGs and DEMGs, rather than the phase-specific results.

To further explore the importance of the matrisome in tissue dysregulation between endometriosis and normal endometrium, we performed functional enrichment analysis on our final phase-union set of DEGs, defined as those genes that were differentially expressed in one or more menstrual phases. We identified several gene ontology (GO) terms (groups of functionally related genes found to be overrepresented among gene sets of interest using enrichment analysis) as enriched among our DEG list (Fig. 3D). [10] Enriched GO terms included functions such as focal adhesion, cell response to extracellular stimulus, and functions related to collagen and other structural ECM composition. These results further supported our decision to narrow our investigation to the matrisome gene expression specifically, instead of global gene expression.

### Unsupervised Analysis

**Fig. 4:**
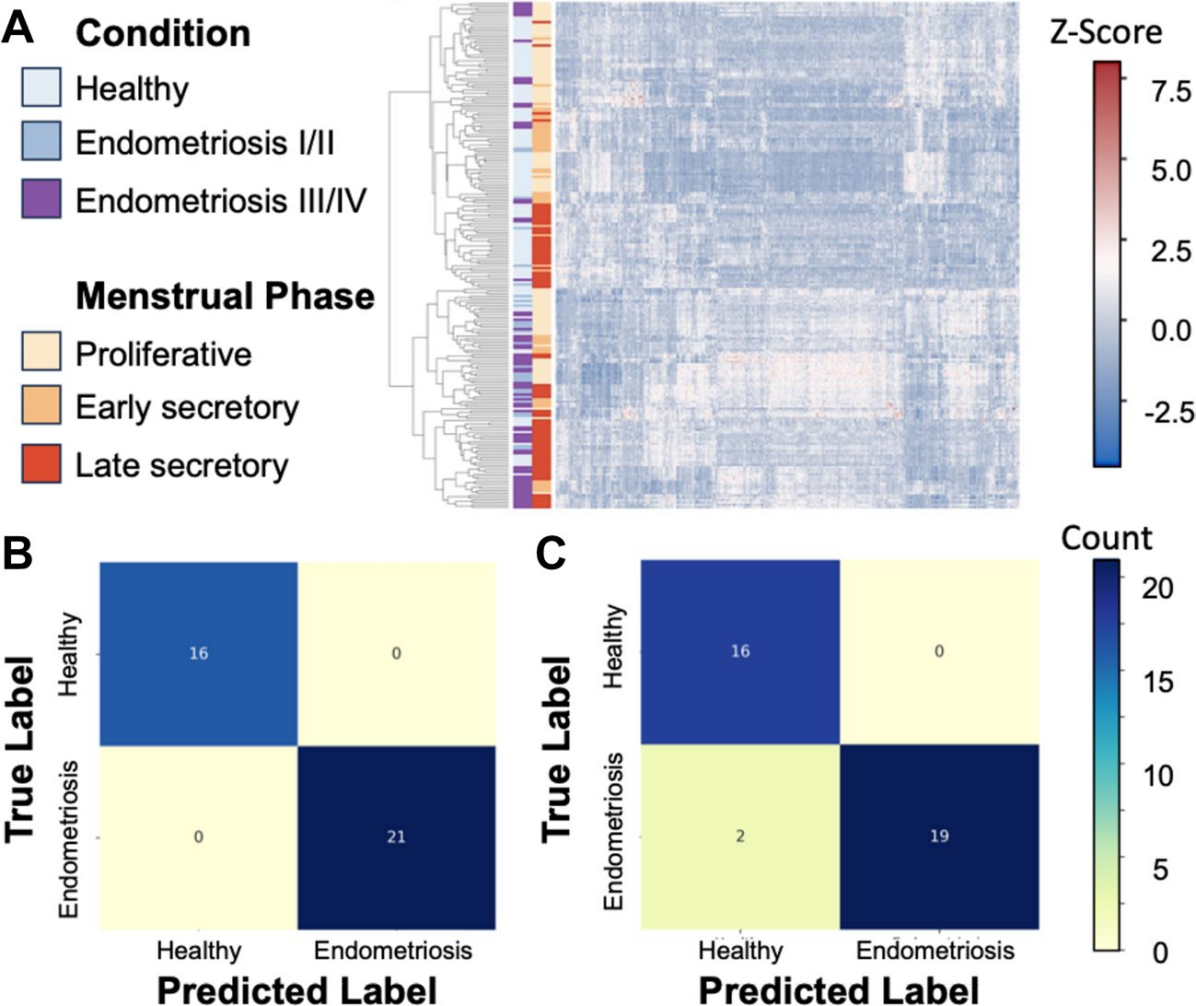
**A** Heatmap and hierarchical clustering of matrisome gene expression levels from normal and endometriosis samples of eutopic endometrium (GSE4888, GSE6364, GSE51981, and GSE29981). Rows represent genes, and columns represent samples. Samples are color coded by condition (healthy, light blue; endometriosis I/II, soft blue; endometriosis III/IVm deep purple) and menstrual phase (proliferative, beige; early-secretory, light orange; late-secretory, dark orange). **B** Confusion matrix depicting the performance of the machine learning classification model using all of matrisome genes. True positives are instances where the samples with a true label of endometriosis was predicted to have a label of endometriosis (21/21), and true negatives are instances where the samples with a true label of healthy were predicted to have a label of healthy (16/16). **C** Confusion matrix depicting the performance of the machine learning classification model only using the core matrisome genes. True positives are instances where the samples with a true label of endometriosis was predicted to have a label of endometriosis (19/21), true negatives are instances where the samples with a true label of healthy were predicted to have a label of healthy (16/16), and false negatives are instances where the samples with a true label of endometriosis was predicted to have a label of healthy (2/21)

To gain insights into the underlying patterns of matrisome gene expression, we conducted hierarchical clustering, an unsupervised approach that allowed samples to group based solely on their matrisome gene expression profiles. Clustering revealed distinct clusters of samples grouped by similarity in matrisome expression (using the Euclidean distance metric). Subsequent examination of the clinical labels associated with each cluster indicated that samples within the same cluster often shared similar clinical conditions and menstrual cycle phases, suggesting a correlation between matrisome gene expression and relevant clinical characteristics (Fig. 4A).

### Machine Learning Classification Between Endometriosis and Normal Tissue, Using Matrisome Gene Expression

We then used machine learning techniques to construct optimized elastic net penalized logistic regression models, aiming to explore the potential of matrisome gene expression to stratify normal and endometriosis samples. We considered two scenarios: using all matrisome genes and using only core matrisome genes [25]. Additionally, since we observed that menstrual phase significantly contributed to the variance in matrisome gene expression, we evaluated model accuracy separated by menstrual phase. When using all matrisome genes, the logistic regression models demonstrated exceptional performance, achieving over 95% accuracy in the training set (*n* = 179) and 100% accuracy on an independent test set (*n* = 37) (Fig. 4B). [26] However, if only core matrisome genes were used, 5% of the samples were misclassified (Fig. 4C). Taken together, these results suggest the expression of the full set of matrisome genes could be used for diagnostic purposes to distinguish endometriosis from normal tissue. Additionally, they reinforce the significance of gene expression alterations within the matrisome in the context of endometriosis.

### Stage Significance Analysis

To explore the dynamics of how the matrisome changes with increasing endometriosis stage, we performed several univariable and multivariable analyses, as well as weighted gene correlation network analysis (WGCNA) on the matrisome genes present in our dataset. Matrisome genes found to be significant via any of these analyses were cross-referenced with the DEMGs and classified using the following terms: DEMGs that were found to be significant via univariable or multivariable analyses were termed *stage model significant*; DEMGs that were significant via WGCNA were deemed *stage network significant*; DEMGs that were both stage model significant and stage network significant were deemed *stage significant*.

### Univariable and Multivariable Analyses to Assess Association with Endometriosis Stage

**Fig. 5:**
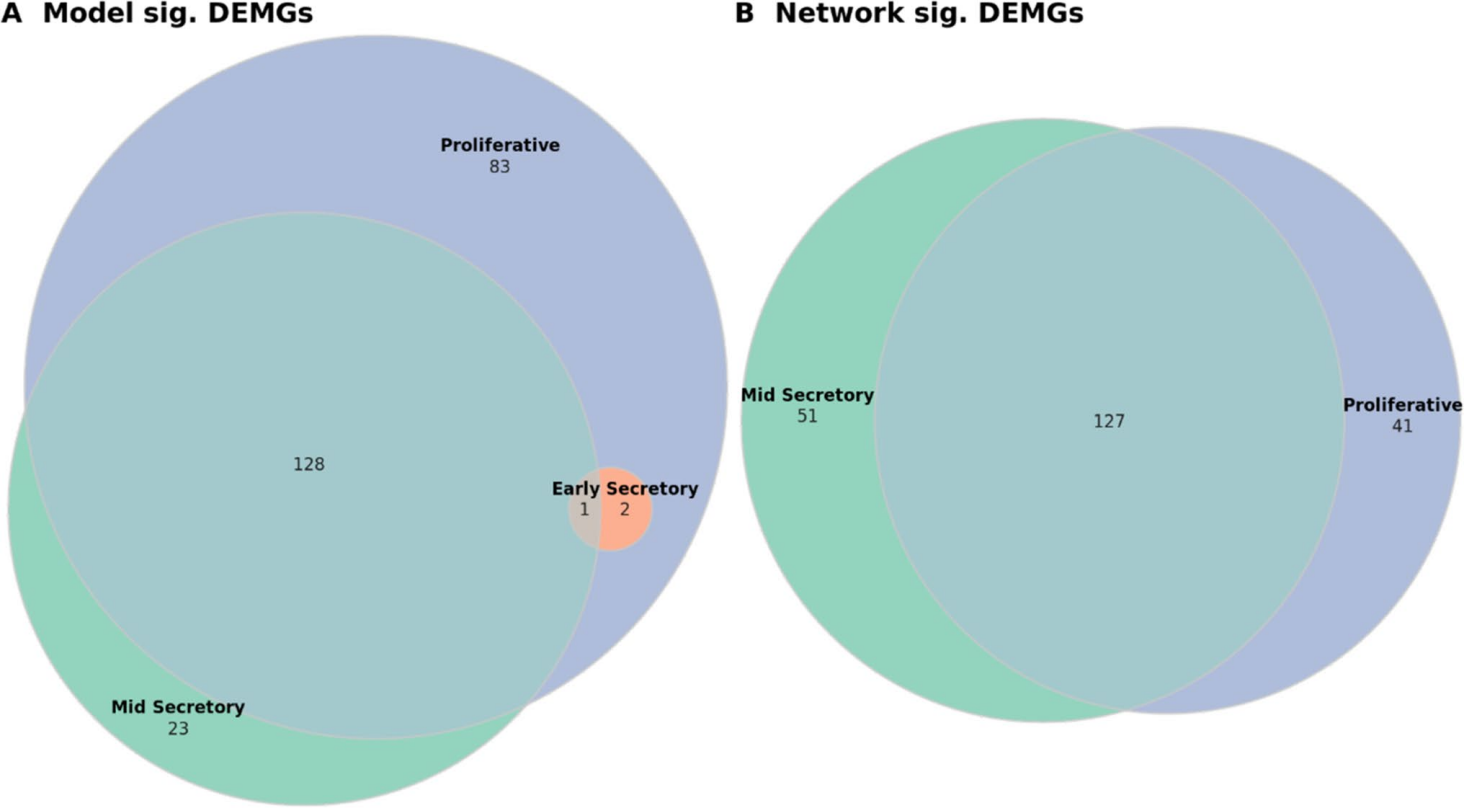
Overlaps among phases with respect to stage model significant DEMGs and stage network significant DEMGs. Overlaps among proliferative, early-secretory, and mid-secretory samples in terms of **A** stage model significant and **B** stage network significant differentially expressed matrisome genes (DEMGs)

To investigate the relationship between individual matrisome genes and endometriosis stage, we performed the following univariable analyses: gene-wise point-biserial correlation tests between each matrisome gene and endometriosis stage [32] and differential gene expression (DGE) analysis among endometriosis samples comparing endometriosis I/II to III/IV. For multivariable analysis, we performed L^1^ penalized logistic regression, classifying endometriosis samples as endometriosis I/II or III/IV [34, 41]. Within each menstrual phase, the L^1^ penalized logistic regression models settled on a similar number of DEMGs, and all models performed reasonably well compared to baseline values (Supplemental Tables 5, 6). For point-biserial correlation and stage-wise DGE analysis, significance was determined based on $q$-values. For L^1^ penalized logistic regression, significance was determined based on non-zero coefficient values. These analyses yielded 214, 3, and 152 unique model significant DEMGs among the proliferative, early-secretory, and mid-secretory samples, respectively (Table 3). Of the 237 unique DEMGs identified as stage model significant within at least one phase, only 23 were not present among proliferative phase samples (Fig. 5) and only one gene, *ANXA4*, had conflicting effects between groups. *ANXA4* was shown to be upregulated in III/IV compared to I/II endometriosis in both proliferative and mid-secretory samples but downregulated in early-secretory samples. All other overlaps among model significant DEMGs between phases agreed in terms of gene effect (point-biserial correlation sign, fold-change sign in I/II and III/IV DGE analysis, or coefficient sign in penalized logistic regression model).

**Table 3:** Stage model, stage network, and stage significant differentially expressed matrisome genes (DEMGs). Number of DEMGs found to be significant with respect to endometriosis stage among models, networks, or both. Results presented within phase and as union of phases. Union of methods is defined as unique genes when method results were pooled (point-biserial correlation, stage-wise differential gene expression (DGE) analysis and penalized logistic regression)

c				
Phase	Point-biseral correlation	Stage-wise DGE analysis		L^1^ penalized logistic regression
Proliferative	203	61		7
Early-secretory	0	1		2
Mid-secretory	132	57		6
Union of phases	227	94		14
Stage model significant DEMGs (union of methods)				
Proliferative	214			
Early-secretory	3			
Mid-secretory	152			
Union of phases	237			
Stage network significant DEMGs				
Phase	Significant modules		Significant genes	
Proliferative	2		168	
Mid-secretory	2		178	
Union of phases	4		219	
Stage significant DEMGs (model or network)				
Phase	Significant genes			
Proliferative	242			
Early-secretory	3			
Mid-secretory	194			
Union of phases	259

### Weighted Gene Correlation Network Analysis

**Fig. 6:**
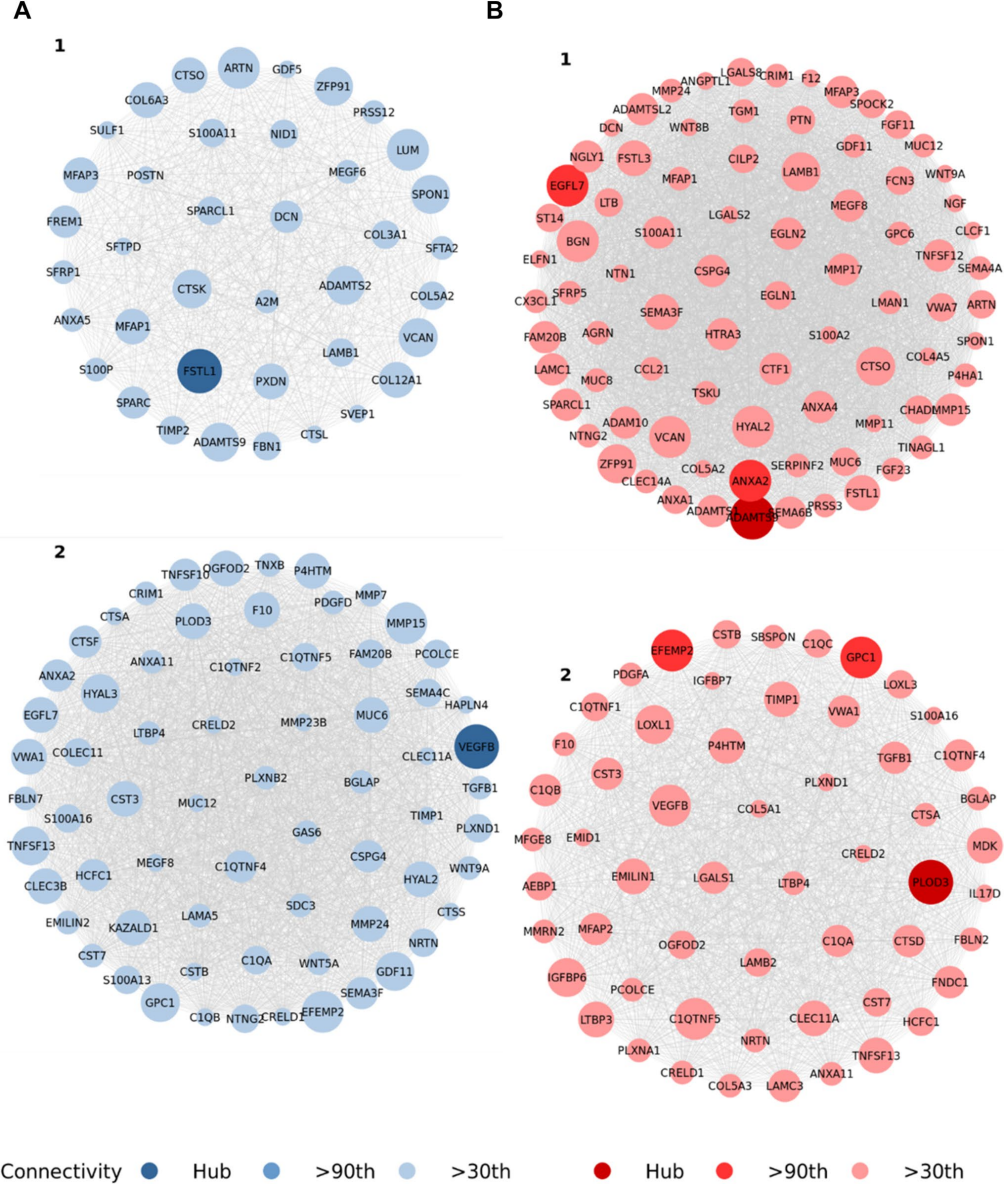
Matrisome gene network modules. Differentially expressed matrisome genes (DEMGs) whose modules, found using weighted gene correlation network analysis (WGCNA), were significantly correlated with endometriosis stage in the **A** proliferative and **B** mid-secretory phases. Module genes were filtered, scaled, and shaded based on connectivity as compared to the connectivity of their module’s hub gene, the most connected DEMG in the module. Module DEMGs which were below the 30^th^ percentile in terms of connectivity are not pictured, but were utilized in our analyses. Module DEMGs which were in the 90^th^ percentile of connectivity are shaded darker than those below the 90^th^ percentile. Hub genes are shaded darkest. Connectivity was determined based on the row-wise (gene-wise) sum of a given module’s adjacency matrix. Connectivity is relative to each module within each cohort, so node sizes cannot be compared between modules within the same or different cohorts

WGCNA analysis identified two significant matrisome gene modules in the proliferative and two significant gene modules in the mid-secretory phases, indicating the presence of co-expressed clusters of matrisome genes within each of these two phases (Fig. 6). Between these four modules, we identified 219 unique network significant DEMGs, with 168 and 178 network significant DEMGs found in the proliferative and mid-secretory phases, respectively. As with all other analyses, extensive overlap was observed between network significant genes in the proliferative and mid-secretory phases. Gene networks were unsigned, so unlike the univariable and multivariable analyses, agreement in terms of effect direction (e.g., up or downregulation) was not assessed. Early-secretory phase samples were explored with WGCNA, but no significant modules were identified, and a reasonable soft threshold value (used in WGCNA to construct topological overlap measure matrix) was not achievable for these samples [42]. Finally, networks within phases were explored to identify hub genes, defined as genes with high levels of connectivity within their respective modules.

### Stage Significant Genes

Both the model and network analyses evaluated gene significance with respect to disease stage. Thus, results for these analyses were combined within each menstrual phase. As with our DGE analysis between diseased and normal endometrium, the majority of stage significant DEMGs across all menstrual phases were a subset of those that were significant within the proliferative phase. Therefore, the DEMGs that were both network and model significant were pooled between phases. Early-secretory had very few stage significant DEMGs, which may be due to reduced statistical power, as there were fewer samples in the early-secretory phase compared to the other menstrual phases (Supplemental Table 8). Alternatively, the smaller number of stage significant DEMGs could be due to an underlying biological mechanism.

**Fig. 7:**
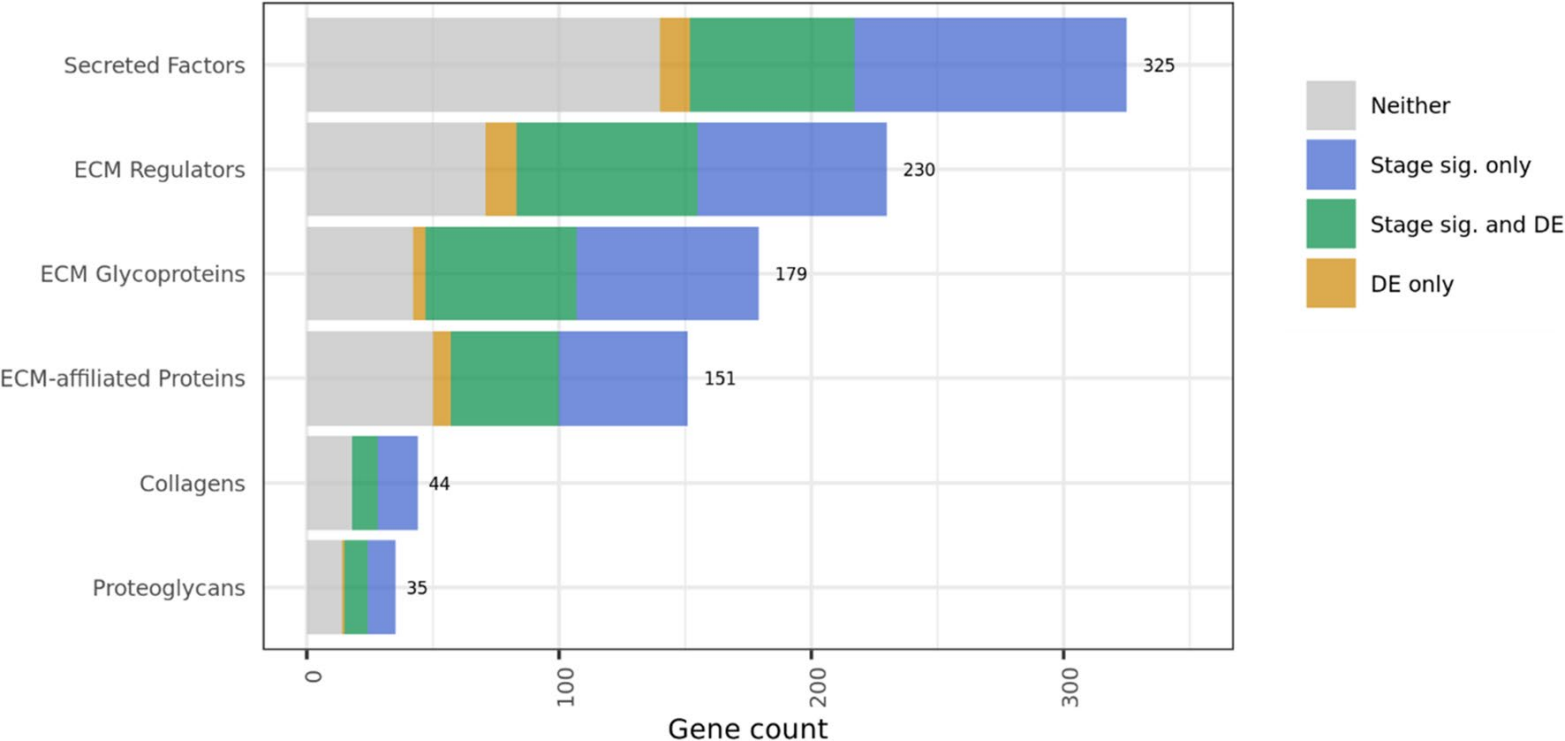
Matrisome category differential expression and stage significance breakdown. Visualization of the overlap between matrisome genes which are differentially expressed (DE) in endometriosis versus normal tissue and matrisome genes which have inferential significance with respect to endometriosis stage. The green area (overlap between DE and stage significant matrisome genes) represents the stage significant differentially expressed matrisome genes which were the subject of a large portion of our analysis

Among the stage significant DEMGs were 60 secreted factors including chemokines *CCL3*, *CCL5*, *CCL14*, *CCL21*, *CX3CL1*, and *CXCL14*, interleukins *IL13, IL15*, and *IL17C*, growth factors *NGF*, *PDGFA*, *TGFB1*, *TNF*, and *VEGFB*, 65 ECM regulators including ADAM metallopeptidase, matrix metallopeptidase, cathepsin, and lysyl oxidase families, 56 glycoproteins including agrin, elastin, fibrillin, laminin, and matrillin families, 41 ECM affiliated proteins including lectin and mucin families, 10 genes related to collagen including the COL4A and COL5A families, and 8 proteoglycans including decorin, podocin, and versican. These genes were again evaluated by matrisome category, and the relative proportions of genes that were differentially expressed only, stage significant and differentially expressed, or stage significant only were visualized for each matrisome category (Fig. 7).

### Functional Enrichment and Pathway Analyses

**Fig. 8:**
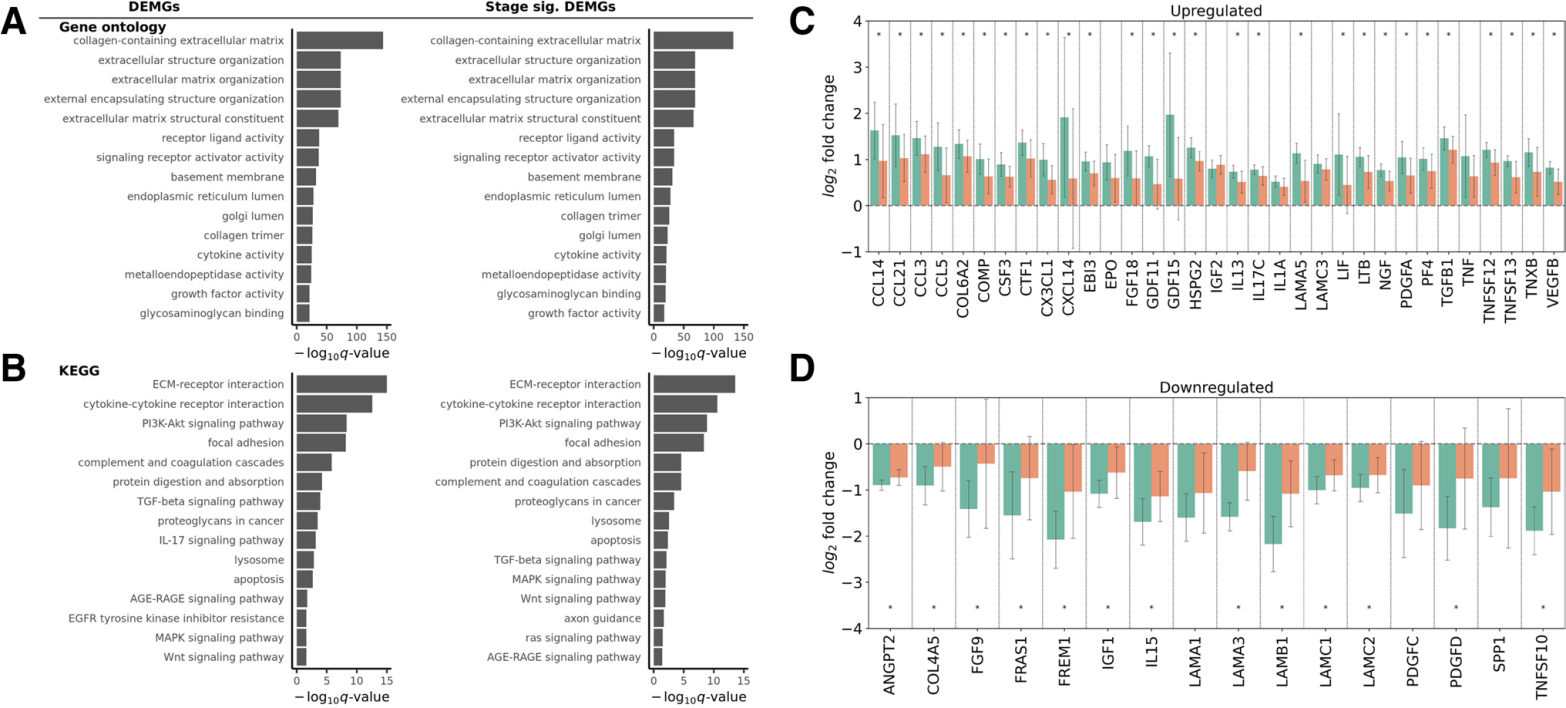
Functional enrichment and pathway analysis. **A** Gene ontology (GO) functional enrichment of stage-significant differentially expressed matrisome genes (DEMGs). Value of $-\left(q\right) >1.3$ indicates significance ($q<0.05$). **B** KEGG pathway analysis results of stage-significant DEMGs. Value of $-\left(q\right) >1.3$ indicates significance ($q<0.05$). **C** Gene expression of DEMGs within the top five significantly enriched KEGG pathways that are upregulated in endometriosis compared to healthy samples. Data are from endometriosis I/II and III/IV samples in the proliferative phase. *N* = 12 proliferative endometriosis stage I/II, *N* = 23 proliferative endometriosis stage III/IV; *p < 0.05 by *t*-test. **D** Gene expression of DEMGs within the top five significantly enriched KEGG pathways that are downregulated in endometriosis compared to healthy samples. Data are from endometriosis I/II and III/IV samples in the proliferative phase. *N* = 12 proliferative endometriosis stage I/II, *N* = 23 proliferative endometriosis stage III/IV; *p < 0.05 by *t*-test

Among stage-significant DEMGs, gene ontology (GO) terms such as extracellular matrix, extracellular structure, and external encapsulating structure organization were highly enriched due to dysregulation of ADAM and ADAMTS family genes, collagens, laminins, matrix metallopeptidases, and others (Fig. 8A). Basement membrane, cytokine activity, growth factor activity, and glycosaminoglycan binding were also significantly enriched stage significant DEMGs (Fig. 8A). The Kyoto Encyclopedia of Genes and Genomes (KEGG) is a knowledge base which connects established genomic information with high order functional behavior among genes, defining pathways which describe critical cellular processes [11]. Pathways that were significantly enriched among stage significant DEMGs included ECM-receptor interaction, cytokine–cytokine receptor interaction, PI3K-Akt signaling, focal adhesion, complement and coagulation cascades, protein digestion and absorption, TGF-β signaling, lysosome, AGE-RAGE signaling, MAPK signaling, Wnt signaling, and axon guidance (Fig. 8B).

We then analyzed gene expression from endometriosis I/II and III/IV samples in the proliferative phase, focusing on genes in the top five significantly enriched KEGG pathways: (1) ECM-receptor interaction, (2) cytokine–cytokine receptor interaction, (3) PI3K-Akt signaling pathway, (4) focal adhesion, and (5) protein digestion and absorption. Interestingly, among the 49 genes involved in these pathways, all but one exhibited greater dysregulation compared to healthy samples in endometriosis I/II compared to endometriosis III/IV. Upon closer examination of the genes upregulated in endometriosis compared to healthy samples, 28 genes had statistically significant increased expression in endometriosis I/II compared to III/IV, while 4 genes followed this trend but were not statistically significant (Fig. 8C). Examining the genes that were downregulated in endometriosis compared to healthy samples, 13 genes had statistically significant decreased expression in endometriosis I/II compared to III/IV, while 3 genes followed this trend but were not statistically significant (Fig. 8D). These patterns were consistent across a broader analysis of all stage significant DEMGs. Specifically, of the 259 stage significant DEMGs, 239 exhibited this same trend of greater dysregulation in endometriosis I/II compared to III/IV, with only 3 genes being more dysregulated in the endometriosis III/IV samples compared to the endometriosis I/II samples. Furthermore, when separate DGE analyses were performed of endometriosis I/II compared to healthy samples and endometriosis III/IV compared to healthy samples, we found that only 7 genes were exclusively upregulated in endometriosis I/II and not III/IV and only 10 genes were exclusively downregulated in endometriosis I/II and not III/IV. This trend further underscored the observation that the matrisome genes were notably more dysregulated in the endometriosis I/II samples compared to the endometriosis III/IV samples.

## Discussion

In summary, we analyzed the relationship between matrisome gene expression and the presence and stage of endometriosis. First, we identified genes that were differentially expressed between endometriosis and normal tissue and established that ECM-related GO terms were significantly enriched among these genes. Next, we demonstrated that machine learning models could accurately distinguish between normal and endometriosis tissue using matrisome gene expression data alone. We then identified matrisome genes and gene networks that had inferential significance to delineate endometriosis stages I/II from III/IV and used these results to identify dysregulated pathways and gene ontology terms.

The endometrium is a highly dynamic tissue, necessitating separate analysis of samples from different menstrual cycle phases. Our results provide valuable insights into the potential significance of the proliferative phase in studying endometriosis. Notably, we observed that dysregulation of matrisome genes in the early- and mid-secretory phases appear to be a subset of the dysregulation observed in the proliferative phase. The proliferative phase samples exhibited the highest number of differentially expressed genes, both overall and within the matrisome gene group. This observation aligns with the proliferative phase’s role in increased endometrial growth and repair, making it inherently relevant to the matrix remodeling associated with endometriosis. Consequently, the proliferative phase could present an opportune time for conducting protein or gene expression-based analysis on tissue from people with endometriosis. Furthermore, our findings suggest that dysregulation of matrisome genes in endometrial tissue from people with endometriosis compared to those without could be used for diagnostic purposes to distinguish endometriosis from normal tissue.

Our work confirmed and consolidated previous findings on the dysregulation of matrisome genes and pathways in endometriosis. For example, similar to previous bioinformatics studies of endometriosis, we found that ECM-receptor interactions, cytokine–cytokine receptor interactions, immune–stromal cell interactions, coagulation cascades, and TGF-β signaling were dysregulated in endometriosis tissue compared to healthy endometrium [7, 43–46]. We also found that inflammatory and neurotransmission cytokines and pathways were correlatively dysregulated, which is in line with studies that have investigated neuroinflammation in endometriosis patients [47, 48]. The PI3K-Akt signaling pathway was significantly enriched in endometriosis samples and inferentially significant for endometriosis stage. Upregulation of PI3K-Akt has been reported in animal models of endometriosis as well as eutopic endometrium samples from people with endometriosis*.* [49, 50] The AGE-RAGE was also dysregulated and significant for endometriosis stage, which has been linked to endometriosis pathogenesis as well as oxidative stress, inflammation, apoptosis, and angiogenesis [51]. Lastly, our work confirmed that both the MAPK and Wnt signaling pathways are highly dysregulated in endometriosis, which have been implicated in endometriosis pathology through *in vitro* experiments. [52, 53]

In an effort to better understand the differences between samples from different endometriosis stages, we observed a surprising trend: the dysregulation of matrisome gene expression was more pronounced in endometriosis I/II samples compared to endometriosis III/IV samples. This was true for 93% of all stage significant DEMGs and 84% of stage significant DEMGs in the top five significantly enriched KEGG pathways. To our knowledge, we are the first to make this observation, which is the opposite of what we anticipated. Future work could expand on this finding.

While combining results from different phases for enrichment analysis was justifiable given the extensive inter-phase overlaps observed, this may obfuscate more granular characteristics of each phase. Additionally, our analyses were limited to only eutopic samples of normal and endometriosis endometrium, thus relying on the retrograde menstruation theory of endometriosis origins [1]. This constraint allowed us to control for variation attributable to the tissue of origin but prevented us from considering matrisome characteristics of ectopic endometrium. As endometriosis datasets grow in size and tissue diversity, matrisome expression analysis of ectopic endometrium could be an area of interest for future work. Future work could also attempt to deconvolve the activity of specific cell types involved in the matrisome dysregulation we observed, similar to the use of CIBERSORT and xCell in the work by Poli-Neto [4, 54, 55]. Finally, we only investigated matrisome genes that were dysregulated between normal and endometriosis tissue overall when assessing genes that held significance for delineating endometriosis I/II from III/IV. Future work could expand on our unfiltered analysis and explore results for matrisome genes which were stage significant without cross-referencing for differential expression in disease overall.

Additionally, we acknowledge several potential limitations inherent in our analytical approach. The use of machine learning models involves adjusting multiple hyperparameters, which could introduce bias and potentially reduce the model’s ability to generalize to new datasets. Although we have carefully optimized these hyperparameters to obtain reliable results, it is essential to be aware of the potential influence on the findings. Moreover, the reliance on traditional statistical cutoffs, such as *p* < 0.05, although widely accepted, may be considered somewhat arbitrary and could be subject to debate. Results that hover around these cutoffs warrant careful interpretation, as different threshold choices could lead to varying conclusions. We have exercised caution in interpreting our results and have taken into account the implications of the selected cutoffs. These limitations do not diminish the value of our study; rather, they underscore the complexities inherent in such analyses and pave the way for future research and potential refinement of our understanding. By being open about these potential biases and limitations, we aim to encourage further investigation and discussion within the scientific community. The consideration of these factors will allow readers to interpret our results with full awareness of possible future, potentially divergent, interpretations.

This work builds upon our previous work that used a similar approach to analyze the relationships between matrisome gene expression and gynecological cancers [56]. This analysis pipeline represents a clear and consolidated application of many of the most influential and well-established methods for analyzing transcriptional data and machine learning methods to evaluate the significance of matrisome genes and gene networks in the context of disease dynamics. This provides individuals with expertise in ECM biology or tissue engineering but little expertise in computer science with an overview of how to analyze their datasets and identify matrisome components of interest for their applications.

Overall, the work presented here is one of the most comprehensive omics analyses of endometriosis data currently available, and to our knowledge, the only such study which focuses on exploring matrisome dysregulation of endometriosis. Our results reinforce and expand upon previous findings related to gene expression dysregulation in endometriosis and hold significant value for future drug discovery and tissue engineering research focused on endometriosis.

### Supplementary Information

Below is the link to the electronic supplementary material.

Supplementary file1 (DOCX 22 KB)

## Data Availability

All data and code are available on the Fogg Lab Github (https://github.com/fogg-lab/)
