# Supplementary material for: Characterizing the Extracellular Matrix Transcriptome of Endometriosis
Source: Reprod Sci. 2023 Oct 3;31(2):413–29. doi: 10.1007/s43032-023-01359-w (PMC10827821; doi:10.1007/s43032-023-01359-w)
Supplement: Supplementary file 1 — Supplementary file1 (DOCX 22 KB) [file 43032_2023_1359_MOESM1_ESM.docx]

**Tables**

| GEO Series | Tissue Source | Condition | Phase | Count of Samples |
| --- | --- | --- | --- | --- |
| GSE4888 | Eutopic endometrium | Healthy | Early Secretory | 3 |
|  |  |  | Mid Secretory | 8 |
|  |  |  | Proliferative | 4 |
| GSE6364 | Uterine fundus | Endometriosis III/IV | Early Secretory | 6 |
|  |  |  | Mid Secretory | 9 |
|  |  |  | Proliferative | 6 |
|  |  | Healthy | Early Secretory | 3 |
|  |  |  | Mid Secretory | 8 |
|  |  |  | Proliferative | 5 |
| GSE51981 | Archived samples from the NIH UCSF Human Endometrial Tissue Bank (eutopic endometrium) | Endometriosis I/II | Early Secretory | 6 |
|  |  |  | Mid Secretory | 9 |
|  |  |  | Proliferative | 12 |
|  |  | Endometriosis III/IV | Early Secretory | 12 |
|  |  |  | Mid Secretory | 19 |
|  |  |  | Proliferative | 17 |
|  |  | Healthy | Early Secretory | 12 |
|  |  |  | Mid Secretory | 22 |
|  |  |  | Proliferative | 35 |
| GSE29981 | Eutopic endometrum | Healthy | Early Secretory | 6 |
|  |  |  | Mid Secretory | 4 |
|  |  |  | Proliferative | 10 |

**Supplemental Table 1.** Summary of tissue source and condition for GEO datasets used in this study.

| ***All genes* (**$\mathbf{n=21,407}$**)** | | | | |
| --- | --- | --- | --- | --- |
|  | | **Dataset count** | **Post-filtration count** | **Retention rate** |
| All genes | | 21,407 | 17,168 | 80% |
| ***Matrisome genes* (**$\mathbf{n=964}$**)** | | | | |
| **Division** | **Category** | **Dataset count** | **Post-filtration count** | **Retention rate** |
| Core matrisome | Collagens | 44 | 36 | 82% |
| Core matrisome | ECM glycoproteins | 179 | 149 | 83% |
| Core matrisome | Proteoglycans | 35 | 27 | 77% |
| Matrisome-associated | ECM regulators | 230 | 189 | 82% |
| Matrisome-associated | ECM-affiliated proteins | 151 | 124 | 82% |
| Matrisome-associated | Secreted factors | 325 | 235 | 72% |
| Combined | | 964 | 760 | 79% |

**Supplemental Table 2.** Gene filtration rates during differential gene expression analysis. Number of genes overall and number of matrisome genes within each matrisome category before and after expression level filtration as part of DGE analysis pipeline.

|  | **Phase** | | |
| --- | --- | --- | --- |
| **Gene ID** | **Proliferative** | **Early secretory** | **Mid Secretory** |
| ***Endometriosis vs. normal differential gene expression analysis (all genes)*** | | | |
| *ATP12A* | Upregulated | Not sig. | Downregulated |
| ***Stage-wise differential gene expression analysis (matrisome)*** | | | |
| *ANXA4* | Upregulated | Downregulated | Upregulated |

**Supplemental Table 3.** Mismatched gene significance between phases. Among genes shown to be differentially expressed between endometriosis and normal tissue, *ATP12A*, a non-matrisome gene, had differing significance between phases. Among differentially expressed matrisome genes (DEMGs) with significance for endometriosis stage, *ANXA4* showed a different form of significance in different phases. Sample sizes: CESC ($n_{normal}=13$, $n_{tumor}=259$), UCEC ($n_{normal}=105$, $n_{tumor}=141$), and UCS ($n_{normal}=105$, $n_{tumor}=47$).

| **Matrisome Category** | **Number DE** | **Dataset count** | **% DE in matrisome category** |
| --- | --- | --- | --- |
| Collagens | 10 | 44 | 23% |
| ECM glycoproteins | 65 | 179 | 36% |
| ECM regulators | 84 | 230 | 37% |
| ECM-affiliated proteins | 50 | 151 | 33% |
| Proteoglycans | 10 | 35 | 29% |
| Secreted factors | 77 | 325 | 24% |

**Supplemental Table 4.** Differentially expressed matrisome genes by matrisome category. Number and percentage of differentially expressed (DE) matrisome genes in each matrisome category, after performing a set union between all phases), along with number of genes in each matrisome category present in the dataset.

| **Cohort** | **n** | **n_endometriosis_** | **n_normal_** | **Balanced accuracy** |
| --- | --- | --- | --- | --- |
| Proliferative | 78 | 29 | 49 | 0.975 |
| Early secretory | 39 | 18 | 21 | 0.967 |
| Mid secretory | 62 | 28 | 34 | 1 |

**Supplemental Table 5.** Classifier performance. 5-fold cross validated balanced accuracy scores for classifiers within each phase which were trained to stratify endometriosis versus normal tissue. A balanced accuracy of 1 means no classification errors were made.

| **Cohort** | **n** | **n_endometriosis_** | **n_normal_** | **Balanced accuracy** |
| --- | --- | --- | --- | --- |
| Proliferative | 11 | 6 | 5 | 1 |
| Early secretory | 9 | 6 | 3 | 1 |
| Mid secretory | 17 | 9 | 8 | 1 |

**Supplemental Table 6.** Classifier performance on the test dataset (GSE6364). Balanced accuracy scores for classifiers within each phase which were trained to stratify endometriosis versus normal tissue. A balanced accuracy of 1 means no classification errors were made.

| **Phase** | **Best score** | $\boldsymbol{n}$ **coefficients best model** | **Used score** | $\boldsymbol{n}$ **coefficients used model** |
| --- | --- | --- | --- | --- |
| Proliferative | 0.36 | 3 | 0.42 | 27 |
| Early secretory | 0.31 | 7 | 0.33 | 10 |
| Mid secretory | 0.05 | 9 | 0.05 | 14 |

**Supplemental Table 7.** Phase-wise comparison of L1 penalized logistic regression models for classifying endometriosis stage. Performance (measured with cross-validated balanced accuracy) of the L^1^ penalized logistic regression models shows that all models outperformed the baseline of 0.5. The mid secretory models performed best. The used models were those models which had the least penalization which performed within 1 standard error of the best performance. The number of coefficients in a given model includes matrisome genes not yet filtered based on DEMG status.

| **Phase** | **Endometriosis status** | $\boldsymbol{n}$ |
| --- | --- | --- |
| Proliferative | Normal | 28 |
|  | Mild endometriosis | 12 |
|  | Moderate/severe endometriosis | 23 |
| Early secretory | Normal | 9 |
|  | Mild endometriosis | 6 |
|  | Moderate/severe endometriosis | 18 |
| Mid secretory | Normal | 20 |
|  | Mild endometriosis | 9 |
|  | Moderate/severe endometriosis | 28 |

**Supplemental Table 8.** Sample counts within each phase stratified by endometriosis status. Number of samples within each of the three menstrual cycle phases in our dataset, stratified by whether the tissue sample is normal or has mild or moderate/severe endometriosis.
